# Immunoisolation of stem cells by simultaneous encapsulation and PEGylation

**DOI:** 10.1007/s40204-018-0084-3

**Published:** 2018-02-19

**Authors:** Roya Ramezanzadeh Andevari, Sameereh Hashemi-Najafabadi, Fatemeh Bagheri

**Affiliations:** 1 Department of Biomedical Engineering, Faculty of Chemical Engineering, Tarbiat Modares University, Tehran, Iran; 2 Department of Biotechnology, Faculty of Chemical Engineering, Tarbiat Modares University, Tehran, Iran

**Keywords:** Microencapsulation, PEGylation, Alginate, Embryonic stem cells (ESCs), Trimethyl chitosan (TMC)

## Abstract

Today, cell therapy is known as an important tool in the treatment of chronic diseases where cells lose their normal function. Immunoisolation systems using microencapsulation or PEGylation have been developed to evade the problem of rejection by the immune system. The aim of the present study was to investigate a combination of microencapsulation and PEGylation methods in coating mouse embryonic stem cells (mESCs) to determine its effect in reducing the host’s immune response. Therefore, methoxy polyethylene glycol (mPEG) binding on alginate–trimethyl chitosan (TMC) microcapsules was investigated using FTIR. Furthermore, survival of the microencapsulated mESCs was confirmed using AO/PI staining and MTT assays. In addition, the effect of mESCs co-cultured with foreign lymphocytes was evaluated. Overall, interleukin-2 (IL-2) secretions as a response of the immune system revealed that mESCs microencapsulation in alginate–TMC–PEG, reduced the immune system response. The results suggested that IL-2 secretion was reduced to 62% at seventh day.

## Introduction

Cell therapy is an effective method in transplantation medicine. It is a multidisciplinary field between immunology, biomaterials and regenerative medicine. The main objective of cell therapy is to replace the function of injured tissues (Hernández et al. 2010). Existing obstacles in obtaining optimum populations of specific cell lines for cell therapy limit the clinical cell transplantation. Stem cells, including pluripotent embryonic stem cells (ESCs), have high potential for cell therapy; subsequently to overcome the existing limitations of donor cells.

Microencapsulation is a process by which the biologically active materials are enclosed within micro-spherical and semi-permeable containers of 0.2–3.0 mm in diameter (Paredes Juarez et al. 2014; Al-Rammah 2014; Azadi et al. 2016). Alginate is an appealing material that has been widely used for cell encapsulation (Wang et al. 2009).

Proliferation of the encapsulated stem cells (SCs) may depend on the polymer concentration and medium condition (Hashemi and Kalalinia 2015). For example, Wang et al. (2009) showed that ESCs encapsulation within 1% (w/v) alginate gel provides optimal conditions for cell proliferation in comparison to a less or more alginate present. Also, they reported that a modified alginate-based 3D strategy supported proliferation and differentiation of the mouse ESCs (mESCs) into pancreatic insulin-producing cells (Wang et al. 2009; Chayosumrit et al. 2010).

Kim et al. modified the culture conditions to enhance the viability of encapsulated human embryonic stem cells (hESCs) in 1.1% calcium alginate. They showed significantly enhanced viability of the encapsulated hESCs (Kim et al. 2013). Moshaverinia et al. (2013) also developed a novel co-delivery system of RGD-coupled alginate hydrogel containing TGF-b1 for microencapsulation of dental mESCs.

The presence of the second layer in multilayered alginate microcapsules, typically using a polycation, such as poly-*l*-lysine (PLL) or chitosan, serves as the second barrier against the host immune system by decreasing the permeability of larger molecules (Tam et al. 2011).

PEGylation, covalent attachment of polyethylene glycol (PEG), is an alternative method for immunoisolation in cell therapy. The effect of PEGylation using activated methoxy PEGs (mPEGs) on the islets of Langerhans has been studied before, though single layer PEGylation cannot fully protect the cells from the host immune system. Nabavimanesh et al. (2015) developed a new design using simultaneous encapsulation and PEGylation to protect the islets.

In the present study, we investigated simultaneous encapsulation and PEGylation, to immunocamouflage ESCs as a source of cell for future differentiation applications. In this approach, trimethyl chitosan (TMC) was used as a polycation, and activated mPEG was attached on the surface of alginate–TMC microcapsules. Finally, the encapsulated cells viability and immunoprotection property of the microcapsules were investigated.

## Materials and methods

### Materials

High G alginate, mPEG–succinimidyl valeric acid (mPEG–SVA) (10 kDa) and acridine orange (AO) were purchased from BDH (UK), Lysan Bio Inc and Merck (Darmstadt, Germany), respectively. TMC was prepared in Biomedical Engineering Department, Tarbiat Modares University. Dulbecco’s modified eagle medium (DMEM), fetal bovine serum (FBS) and fetal calf serum (FCS) were obtained from Gibco (Carlsbad, CA, USA). RPMI-1640 medium, trypsin/EDTA, and penicillin/streptomycin antibiotics were prepared from Gibco. *l*-glutamine, ß-mercaptoethanol and leukemia inhibitory factor were purchased from Invitrogen, Sigma and Chemicon, respectively. Mouse IL-2 kit was acquired from e-Bioscience (San Diego, CA, USA).

## Methods

### Cell-free microcapsule preparation

Alginate microcapsules were prepared based on Lim and Sun’s method (Sun 1988), and has been explained in our previous work (Nabavimanesh et al. 2015). The alginate beads were transferred to TMC solution (1% w/v in saline) for 10 min, and the prepared microcapsules were washed twice in saline. Finally, the obtained microcapsules were PEGylated using mPEG–SVA, according to our previous work (Nabavimanesh et al. 2015).

### Fourier transform infrared analysis (FTIR)

The presence of TMC and mPEG on the alginate microcapsules was evaluated using FTIR spectroscopy (Perkin Elmer, 500–4000 cm^−1^).

### Cell culture

Rat mesenchymal stem cells (rMSCs) were used as model cells in some early experiments due to some limitations in obtaining ESCs. Bone marrow was extracted from the femur of 8- to 10-week-old male Wistar rats, and was established in DMEM medium containing 15% FBS and 100 U/mL penicillin/streptomycin. The culture was then refreshed twice weekly. At this time, the cells were lifted by trypsin/EDTA and split into two 75 cm^2^ flasks containing fresh medium. In further successive subcultures, rMSCs population was increased.

mESCs were purchased from Royan Institute and maintained at undifferentiated state in gelatin-coated T-25 flasks in DMEM containing 10% FCS, 2 mM l-glutamine, 1% penicillin/streptomycin (100 U/mL/0.1 mg/mL), 0.1% ß-mercaptoethanol and 1000 U/mL leukemia inhibitory factor.

### Encapsulation of SCs

The alginate solution was prepared by dissolving 2 g alginate in 100 mL of Ca^2+^ free DMEM. Adherent SCs were removed following trypsin incubation, and the cell number and viability were investigated using trypan blue exclusion (Wang et al. 2009). According to previously described method, mESCs at a density of 2 × 10^6^ cells per mL of alginate solution were encapsulated in alginate–TMC–PEG. The entire process of encapsulation was performed under sterile conditions.

### Alginate beads solubilization and cell recovery

To determine the average number of cells per capsule, a sample of the prepared capsules was incubated with a solution containing 50 mM sodium citrate, 10 mM glucose and 27 mM NaCl for 30 min at 37 °C. The suspension was centrifuged at 1200 rpm for 6 min, and the sodium citrate solution was aspirated. The pellet was then washed twice with PBS buffer, and re-suspended in DMEM medium for cell counting by trypan blue exclusion (Wang et al. 2009).

### rMSCs viability and proliferation

Viability and proliferation of the encapsulated cells were investigated using MTT assay. MTT assay was performed after 1, 3, 5 and 7 days of culture with three replicates. A sample of 500 µL was removed from each well and replaced with fresh medium. One hundred microliter of MTT solution (5 mg/mL in PBS) was added immediately to each sample. The microcapsules were incubated in MTT-containing medium for 4 h at 37 °C under 5% CO_2_. After incubation, the medium was removed completely, and 500 µL of dimethyl sulfoxide (DMSO) was added into each sample to dissolve the formazan crystals. Absorbance of the obtained solution was measured using a microplate reader at 545 nm.

### Lymphocytes isolation

The lymphocytes were isolated from male C57B1/6 mice (12 weeks old, 25–30 g in weight), according to our previous work (Nabavimanesh et al. 2015).

### Studying the immunological reactions

The immunological reactions were assessed by co-culturing approximately 10^6^ encapsulated or free mESCs with 5 × 10^5^ lymphocytes in each well of a 24-well plate in 1 mL of DMEM/F12 medium under 5% CO_2_ and 37 °C up to 7 days. IL-2 secretion by the lymphocytes co-cultured with ESCs encapsulated in alginate, alginate–TMC, and alginate–TMC–mPEG microcapsules, was assessed according to our previous work (Nabavimanesh et al. 2015).

### mESCs viability after co-culturing with lymphocytes

mESCs viability was determined by staining with AO/PI. The stained encapsulated mESCs in alginate, alginate–TMC, and alginate–TMC–mPEG microcapsules, were assessed using fluorescent microscopy (IX71 OLYMPUS) 7 days after co-culturing with lymphocytes. The mESCs in microcapsules showing green are considered as viable cells.

## Results

### FTIR analysis

**Fig. 1:**
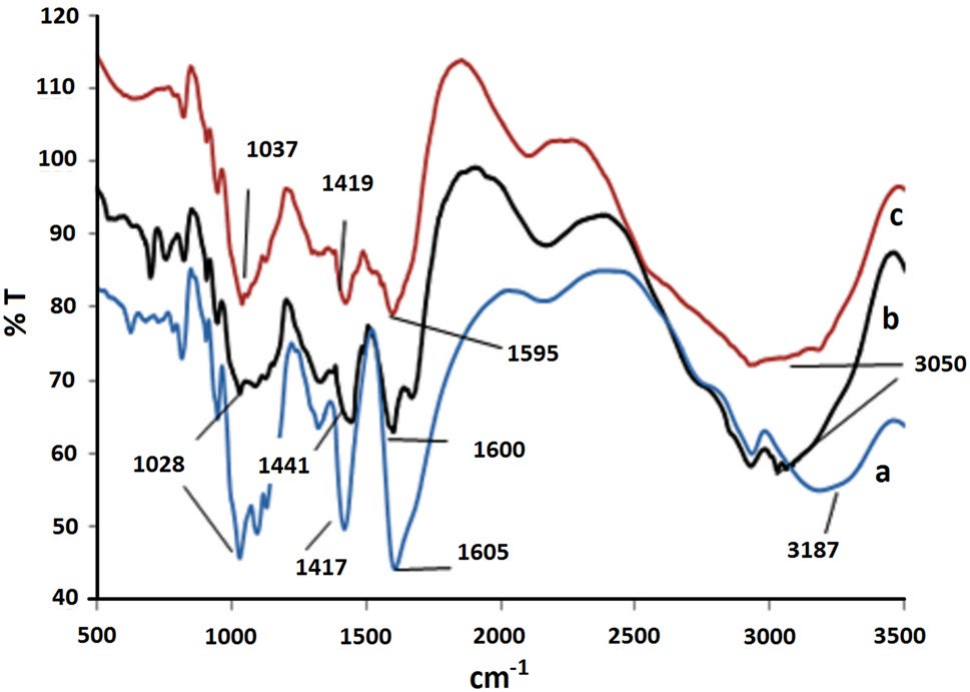
FTIR spectra for alginate (**a**), alginate–TMC (**b**), and alginate–TMC–mPEG (**c**) microcapsules

FTIR spectra obtained for alginate, alginate–TMC, and alginate–TMC–mPEG microcapsules are shown in Fig. 1. It shows that there are several differences between the spectra of alginate, alginate–TMC and alginate–TMC–mPEG microcapsules (in 1000–1200 cm^−1^, 1300–1500 cm^−1^, 1500–1700 cm^−1^ and 3000–3500 cm^−1^ regions) that confirm the attachment of TMC and mPEG on the alginate and alginate–TMC microcapsules, respectively.

### Bead solubilization and cell recovery

After solubilization of alginate microcapsules, the obtained cells were counted using trypan blue exclusion. rMSCs at a density of 1 × 10^6^ cell/mL in alginate solution were encapsulated in three repetitions, and the obtained results showed a cell loading of about 237 ± 7 cells per capsule.

### Cells viability and proliferation

**Fig. 2:**
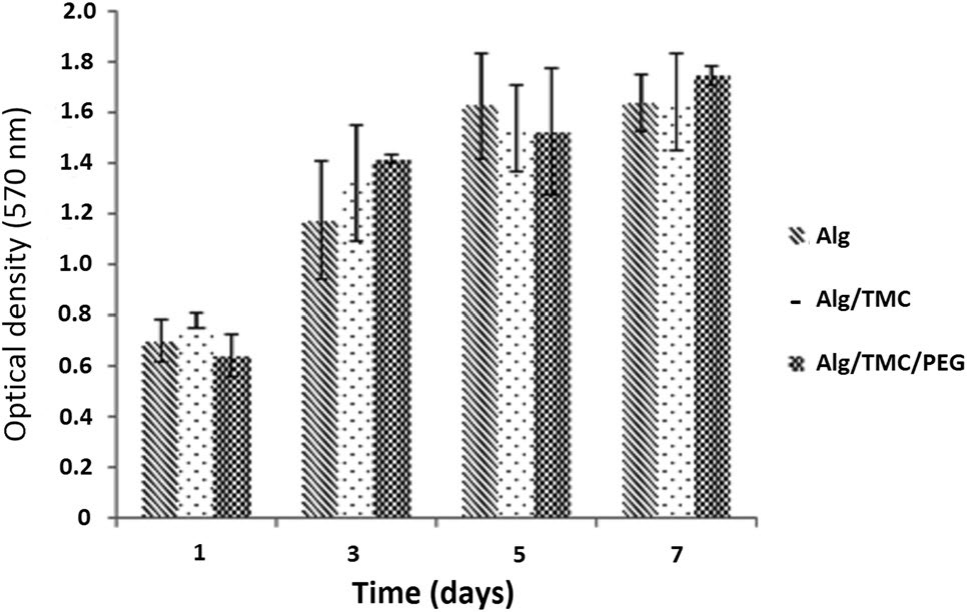
MTT results for the cells encapsulated in alginate, alginate–TMC, and alginate–TMC–PEG microcapsules

Proliferation and viability of the encapsulated cells were measured using MTT assay. Proliferation of MSCs after 1, 3, 5 and 7 days of culture is shown in Fig. 2. MTT assay results indicated that three layers of alginate, TMC and mPEG are biocompatible for MSCs. As it can be seen, MSCs show their highest level of proliferation after 5 days.

### Immunological reactions against the mESCs

**Fig. 3:**
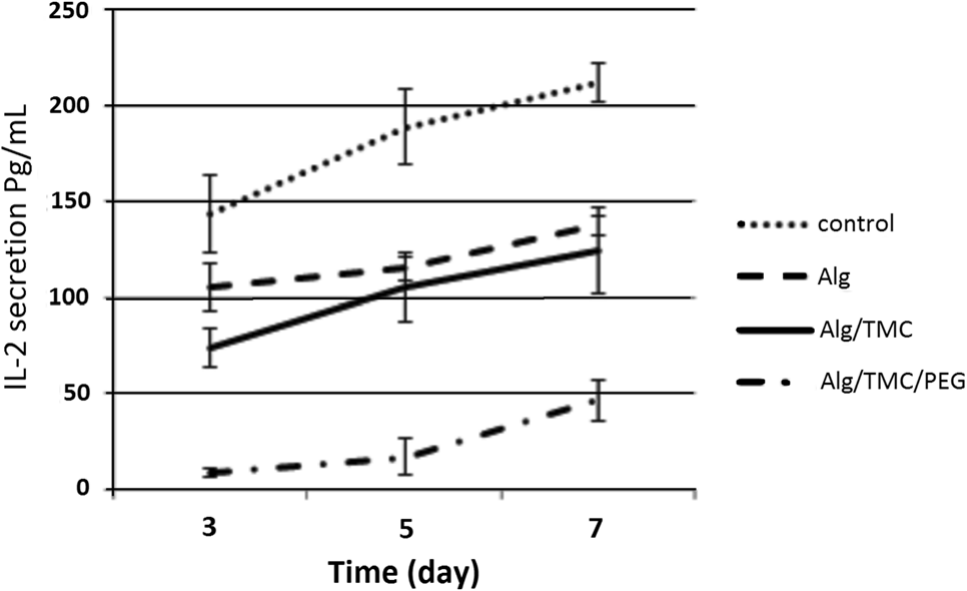
Immunological response against the free and encapsulated mESCs based on IL-2 secretion from the lymphocytes, after 3, 5, and 7 days of co-culturing with free mESCs, and mESCs encapsulated in alginate, alginate–TMC and alginate–TMC–mPEG–SVA microcapsules, separately

Figure 3 shows IL-2 secretion from the lymphocytes against the mESCs in alginate, alginate–TMC and alginate–TMC–mPEG–SVA microcapsules. Every experiment was replicated twice. According to Fig. 3, IL-2 secretion increased overtime in all the samples.

The lymphocytes co-cultured with mESCs encapsulated in alginate showed less IL-2 secretion compared to one with free mESCs, due to the formation of an immunological barrier. On the 7th day, this response decreased by 35% in comparison with free mESCs. TMC addition on the alginate microcapsules decreased IL-2 secretion against the mESCs in comparison with free mESCs and also, it was 9.4% less than the secretion against the mESCs in alginate microcapsules on the 7th day.

As shown in Fig. 3, grafting mPEG–SVA (0.1% w/v) on the surface of alginate–TMC microcapsules also reduced the IL-2 secretion. Therefore, PEGylation prevents the immune cells and improves the immune protection. Moreover, the extent of IL-2 secretion against the mESCs in alginate–TMC–mPEG microcapsules over time was less than that for mESCs in microcapsules without mPEG. For mESCs in alginate–TMC–mPEG–SVA microcapsules; IL-2 secretion was reduced by 62% in comparison with the mESCs in alginate–TMC on the 7th day.

### mESCs viability after co-culturing with lymphocytes

**Fig. 4:**
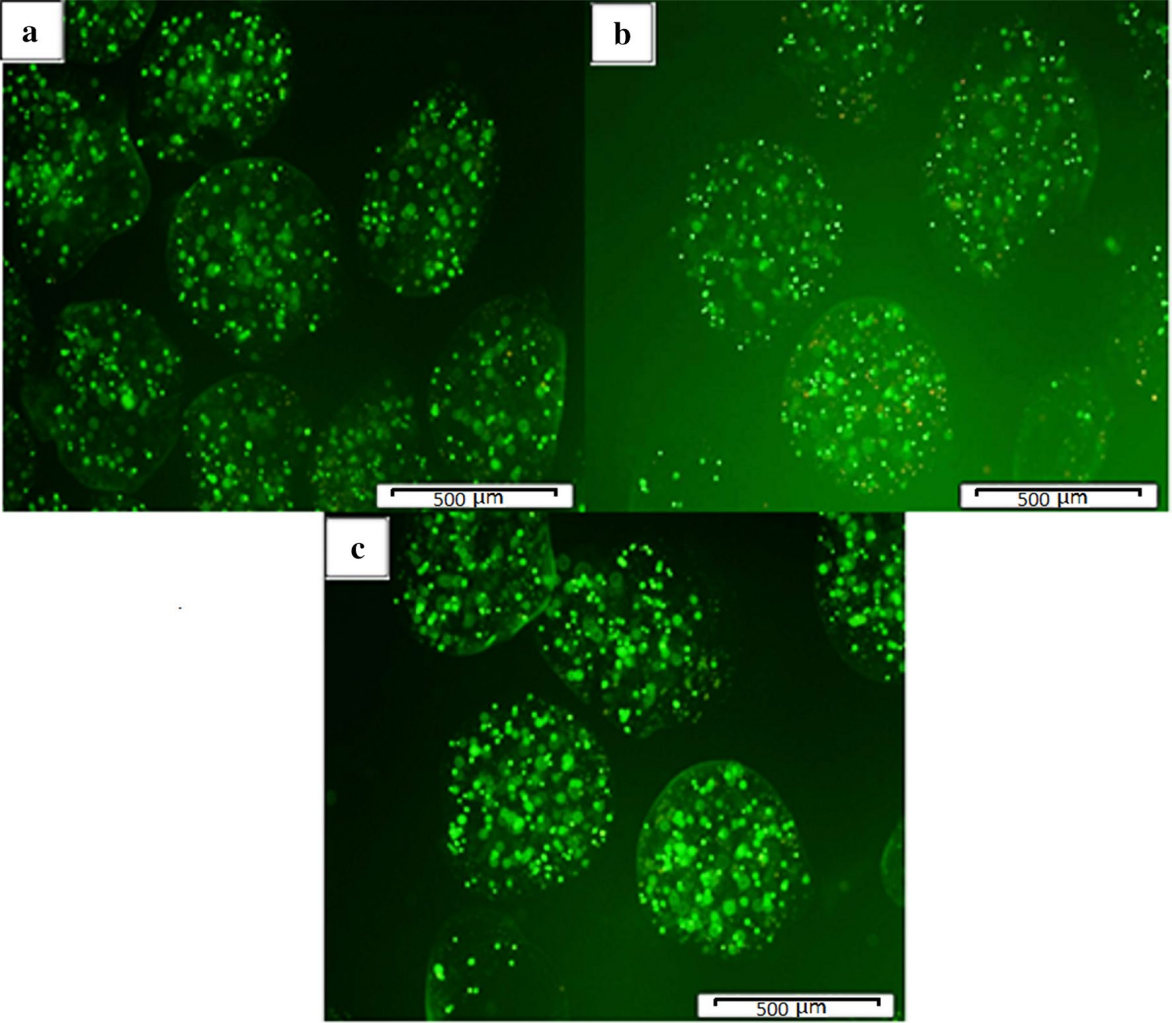
Viability of the encapsulated mESCs in alginate (**a**), alginate–TMC (**b**), alginate–TMC–PEG (**c**) microcapsules, after co-culturing with lymphocytes; using AO/PI assay (scale bar = 500 µm)

Viability of the free mESCs and encapsulated mESCs in alginate, alginate–TMC, and alginate–TMC–mPEG microcapsules was examined after 7 days of co-culturing with lymphocytes. Figure 4 demonstrates that mESCs were viable for 7 days after encapsulation.

## Discussion

Encapsulation and PEGylation are two different methods of immunoisolation in cell transplantation. They have been used simultaneously for islets immunocamouflaging for the first time in our group (Nabavimanesh et al. 2015). The present study investigated simultaneous encapsulation and PEGylation for entrapment of mESCs and we used ESCs to overcome the existing limitations in donor cells.

FTIR spectra of C–O–C bond at 1030 cm^−1^ for alginate, alginate–TMC, and alginate–TMC–PEG microcapsules are related to alginate (Coates 2000; Hu et al. 2013). The observed peak at 1441 cm^−1^ in alginate–TMC spectrum is related to N–CH_3_ bond of amino group that indicates the presence of TMC layer. The angular deformation of N–H bond of amino groups occurs at 1600 cm^−1^ (1500–1620 cm^−1^), though it is weaker due to *N*-methylation in TMC (Mourya and Inamdar 2009). Intensity of the peak at 1030 cm^−1^ increased in alginate–TMC–mPEG microcapsules due to grafting mPEG–SVA on alginate–TMC microcapsules. The observed peak at 1600 cm^−1^ is associated with the presence of amino I group (bending bond) (Sadeghi et al. 2004). The reduced peak intensity for alginate–TMC–mPEG microcapsules, in comparison with alginate–TMC, could be related to the bonding of NH_3_^+^ groups of TMC to COO^−^ groups of mPEG. Additionally, the observed peak at 3000–3500 cm^−1^ is related to hydroxyl and stretching amino groups. Figure 1 shows that addition of a layer to the microcapsules would result in a wider peak in this range. This clearly indicates the presence of TMC on alginate–TMC microcapsules and mPEG on alginate–TMC–mPEG microcapsules corresponding to their amino groups (Coates 2000; Trif and Socaciu 2007; Pavia et al. 2008).

MTT results suggested that encapsulation of the cells in alginate, alginate–TMC and alginate–TMC–mPEG capsules allows cell growth, proliferation and long-term survival of stem cells (Hashemi and Kalalinia 2015). Furthermore, according to Fig. 2, the viable cells were constant on days 5 and 7 after encapsulation. Low space for the proliferation of cells may lead to decreasing in cell growth rate. Thus, the 5th day after microencapsulation may be the optimal time for cell differentiation induction in future works.

To investigate three-layered microcapsules in mESCs immunocamouflaging, specifically, IL-2 secretion against the encapsulated mESCs in alginate, alginate–TMC and alginate–TMC–mPEG microcapsules was measured. IL-2 secretion increasing over time showed that lymphocytes recognized the mESCs during the incubation time. Nevertheless, encapsulation of mESCs prevented direct contact between mESCs and lymphocytes, and as a result it hindered the immune system stimulation. Here, we have demonstrated that mPEG attachment to the surface of alginate–TMC microcapsules decreased the immunological response. Therefore, recognition of the mESCs and IL-2 secretion was reduced.

In cell encapsulation, the cells are immobilized inside a semi-permeable matrix to protect the cells. Other potential advantages of this method may include cell expansion and self-renewal potency or direct cell differentiation toward a desired lineage. Consequently, cell encapsulation is considered as an attractive method for many clinical applications.

## Conclusion

mESCs were encapsulated in alginate, alginate–TMC and alginate–TMC–mPEG microcapsules, separately. A three-layer encapsulation of mESCs prevented direct contact between mESCs and lymphocytes and it hindered their recognition by the immune system. Subsequently, the capsules could adequately protect the cells against the co-cultured lymphocytes at this stage. It also allowed the cell growth and proliferation. In our previous work, we introduced encapsulation and PEGylation system for islets transplantation simultaneously. Here, we proposed it for stem cell capsulation for future works that stem cells should be differentiated to a specific cell line such as bone or insulin-producing cells, inside the capsules and then transplanted.
